# Supplementary material for: Monovalent Salt and pH-Stimulated Gelation of Scallop (Patinopecten yessoensis) Male Gonad Hydrolysates/κ-Carrageenan
Source: Foods. 2023 Sep 28;12(19):3598. doi: 10.3390/foods12193598 (PMC10572707; doi:10.3390/foods12193598)
Supplement: Supplementary file 1 [file foods-12-03598-s001.zip › foods-2569738-supplementary.pdf]

Table S1 T_2 relaxation time curves of SMGHs/KC gels subjected to pH 2-8 and 0.2 M NaCl-stimuli-response.

pH	Relaxation time (*ms*)	
	T_{21} (*ms*)	T_{23} (*ms*)
8	0.58 ± 0.06^a	966.40 ± 39.17^a
5	0.64 ± 0.11^a	1035.88 ± 41.99^a
2	0.49 ± 0.03^a	365.64 ± 14.82^b

Data were expressed as means±SD from triplicate determinations. Different letters in the same column indicated significant differences ($P < 0.05$).

Table S2 T_2 relaxation time curves of SMGHs/KC gels subjected to pH 3-9 and 0.2 M KCl-stimuli-response.

pH	Relaxation time (*ms*)	
	T_{21} (*ms*)	T_{23} (*ms*)
9	0.70 ± 0.12^a	622.26 ± 0.00^a
6	0.62 ± 0.07^a	1086.11 ± 75.36^b
3	0.53 ± 0.19^a	276.98 ± 11.23^c

Data were expressed as means±SD from triplicate determinations. Different letters in the same column indicated significant differences ($P < 0.05$).
